# Human electronegative LDL induces mitochondrial dysfunction and premature senescence of vascular cells in vivo

**DOI:** 10.1111/acel.12792

**Published:** 2018-06-19

**Authors:** Yu‐Chen Wang, An‐Sheng Lee, Long‐Sheng Lu, Liang‐Yin Ke, Wei‐Yu Chen, Jian‐Wen Dong, Jonathan Lu, Zhenping Chen, Chih‐Sheng Chu, Hua‐Chen Chan, Taha Y. Kuzan, Ming‐Hsien Tsai, Wen‐Li Hsu, Richard A. F. Dixon, Tatsuya Sawamura, Kuan‐Cheng Chang, Chu‐Huang Chen

**Affiliations:** ^1^ Division of Cardiovascular Medicine Asia University Hospital Taichung Taiwan; ^2^ Department of Biotechnology Asia University Taichung Taiwan; ^3^ Division of Cardiovascular Medicine China Medical University Hospital Taichung Taiwan; ^4^ Department of Medicine Mackay Medical College New Taipei City Taiwan; ^5^ Cardiovascular Research Laboratory China Medical University Hospital Taichung Taiwan; ^6^ Graduate Institute of Biomedical Materials and Tissue Engineering College of Biomedical Engineering Taipei Medical University Taipei Taiwan; ^7^ International Ph.D. Program in Biomedical Engineering College of Biomedical Engineering Taipei Medical University Taipei Taiwan; ^8^ Department of Radiation Oncology Taipei Medical University Hospital Taipei Taiwan; ^9^ Translational Laboratory Department of Medical Research Taipei Medical University Hospital Taipei Taiwan; ^10^ Department of Medical Laboratory Science and Biotechnology College of Health Sciences Kaohsiung Medical University Kaohsiung Taiwan; ^11^ Lipid Science and Aging Research Center Kaohsiung Medical University Kaohsiung Taiwan; ^12^ Graduate Institute of Biomedical Sciences China Medical University Taichung Taiwan; ^13^ Department of Neuro‐Oncology The University of Texas MD Anderson Cancer Center Houston Texas; ^14^ Vascular and Medicinal Research Texas Heart Institute Houston Texas; ^15^ Department of Surgery The University of Texas Medical Branch Galveston Texas; ^16^ Center for Lipid Biosciences Kaohsiung Medical University Hospital Kaohsiung Taiwan; ^17^ Division of Cardiology Department of Internal Medicine Kaohsiung Medical University Hospital Kaohsiung Taiwan; ^18^ Department of Radiology Marmara University Medical School Istanbul Turkey; ^19^ Department of Physiology Shinshu University School of Medicine Matsumoto, Nagano Japan; ^20^ Graduate Institute of Medicine College of Medicine Kaohsiung Medical University Kaohsiung Taiwan

**Keywords:** atherosclerosis, DNA damage response, electronegative lipoproteins, mitochondria, premature senescence, telomerase

## Abstract

Dysregulation of plasma lipids is associated with age‐related cardiovascular diseases. L5, the most electronegative subfraction of chromatographically resolved low‐density lipoprotein (LDL), induces endothelial dysfunction, whereas the least electronegative subfraction, L1, does not. In this study, we examined the effects of L5 on endothelial senescence and its underlying mechanisms. C57B6/J mice were intravenously injected with L5 or L1 (2 mg kg^−1^ day^−1^) from human plasma. After 4 weeks, nuclear γH2AX deposition and senescence‐associated β‐galactosidase staining indicative of DNA damage and premature senescence, respectively, were increased in the aortic endothelium of L5‐treated but not L1‐treated mice. Similar to that, in Syrian hamsters with elevated serum L5 levels induced by a high‐fat diet, nuclear γH2AX deposition and senescence‐associated β‐galactosidase staining were increased in the aortic endothelium. This phenomenon was blocked in the presence of N‐acetyl‐cysteine (free‐radical scavenger) or caffeine (ATM blocker), as well as in lectin‐like oxidized LDL receptor‐1 (LOX‐1) knockout mice. In cultured human aortic endothelial cells, L5 augmented mitochondrial oxygen consumption and mitochondrial free‐radical production, which led to ATM activation, nuclear γH2AX deposition, Chk2 phosphorylation, and TP53 stabilization. L5 also decreased human telomerase reverse transcriptase (hTERT) protein levels and activity. Pharmacologic or genetic manipulation of the reactive oxygen species (ROS)/ATM/Chk2/TP53 pathway efficiently blocked L5‐induced endothelial senescence. In conclusion, L5 may promote mitochondrial free‐radical production and activate the DNA damage response to induce premature vascular endothelial senescence that leads to atherosclerosis. Novel therapeutic strategies that target L5‐induced endothelial senescence may be used to prevent and treat atherosclerotic vascular disease.

## 1 INTRODUCTION

Aging is an independent risk factor for atherosclerosis (Lakatta & Levy, 2003). With aging, the atheroprotective capability of the arterial endothelium decreases. This change in the endothelium is associated with cellular senescence, which is a stress response characterized by irreversible cell cycle exit and an altered secretory proteome profile (Coppe, Desprez, Krtolica, & Campisi, 2010). Well‐known molecular triggers for the senescence response include attrited telomeres, exposure to sublethal genotoxic stress, and activation of the DNA damage response (DDR) or oncogene signals (Imanishi, Hano, Sawamura, & Nishio, 2004; Mistriotis & Andreadis, 2017; Niemann et al., 2011). In addition, traditional cardiovascular risk factors have been shown to induce premature cardiovascular senescence (Erusalimsky, 2009). In vascular cells, the senescence response is believed to promote atherogenesis via the release of pro‐inflammatory cytokines and decreased nitric oxide production (Wang & Bennett, 2012). In preclinical models, the genetic or pharmacologic blockade of the senescence response has been shown to slow the progression of atherosclerosis (Mercer, Gray, Figg, Kumar, & Bennett, 2012; Zhan, Suzuki, Aizawa, Miyagawa, & Nagai, 2010). Thus, characterizing the intracellular signaling involved in cellular senescence induced by cardiovascular risk factors may provide novel targets for the treatment of atherosclerotic vascular diseases.

Oxidized LDL is an atherogenic lipoprotein that has been shown to accelerate endothelial progenitor cell senescence (Imanishi et al., 2004). However, the quantification of oxidized LDL is controversial and has never been standardized for clinical purposes. In contrast, electronegative LDL is a class of naturally occurring lipoproteins that are as atherogenic as oxidized LDL. The most electronegative subfraction of LDL can be obtained using fast‐protein liquid chromatography with anion‐exchange columns to fractionate human LDL into five subfractions with increasing electronegativity, called L1 to L5 (Chen et al., 2003; Ke et al., 2011). The most electronegative subfraction, L5, is the only LDL subfraction that can induce endothelial dysfunction and atherogenic responses in cultivated arteries and cultured vascular cells or impair normal differentiation of endothelial progenitor cells (Chen et al., 2007; Chu et al., 2013; Lu et al., 2008; Stancel et al., 2016). These biochemical properties of L5 have been attributed to its lipid and protein composition, enzymatic activities, and structural features (Ke, Stancel, Bair, & Chen, 2014; Ke et al., 2016).

We have previously shown that L5 can induce endothelial cell apoptosis, which may contribute to the pathogenesis of acute coronary syndrome. In addition, serum levels of L5 were found to be significantly increased in patients with ST‐elevation myocardial infarction or stroke (Chan et al., 2013; Chang et al., 2013; Shen et al., 2015). In this study, we have further examined the biologic effects of L5 and have explored the possible involvement of L5 in premature vascular endothelial senescence.

## 2 RESULTS

### 2.1 Both exogenously injected L5 and endogenously elevated plasma L5 levels induce endothelial oxidative stress, the DDR, and cellular senescence in vivo

**Figure 1:**
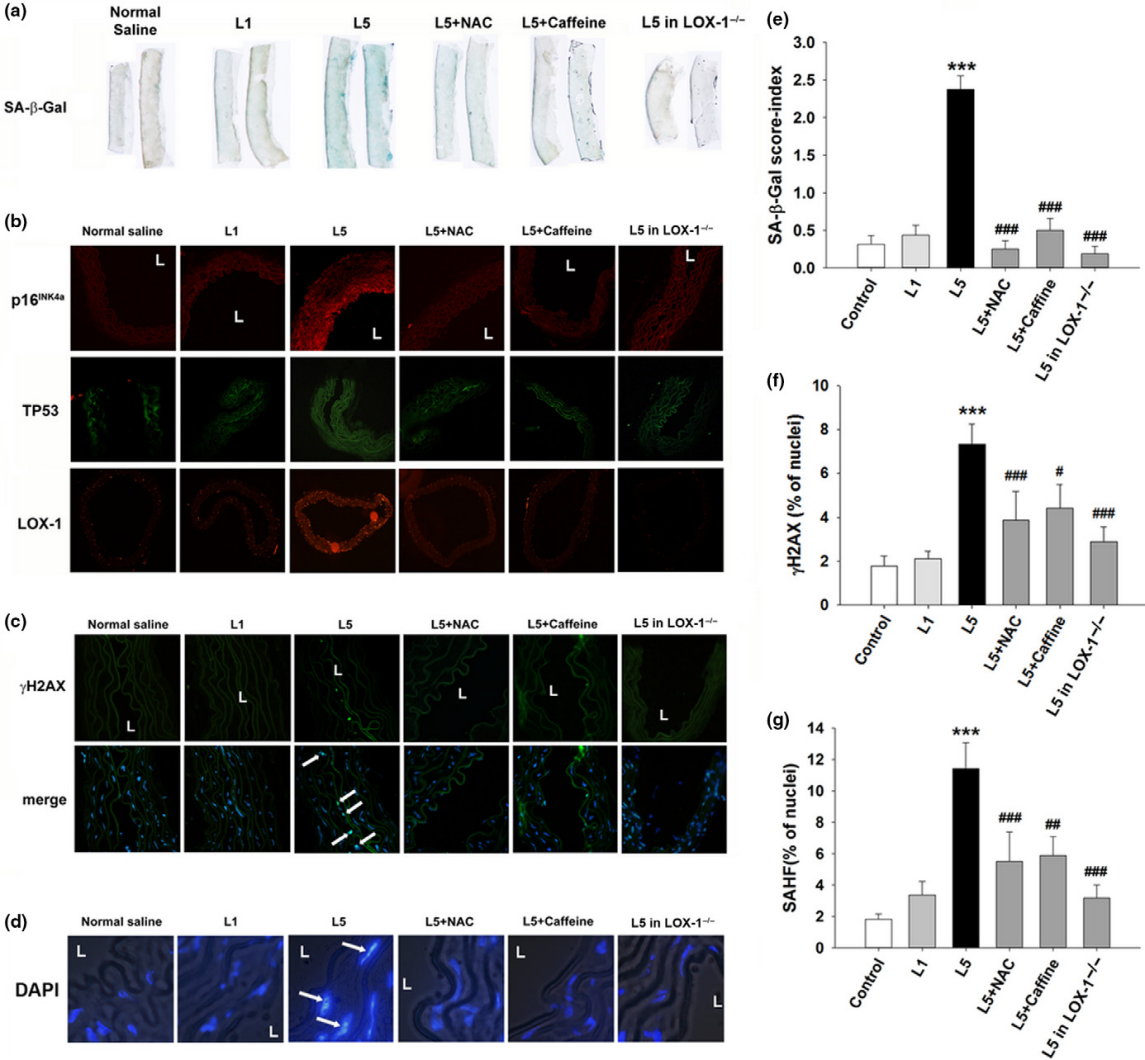
Prosenescent effect of exogenous L5 in vivo. C57B6/J or LOX‐1^−/−^ mice were treated with normal saline, L1 (2 mg kg^−1^ day^−1^), L5 (2 mg kg^−1^ day^−1^), L5+NAC (N‐Acetyl‐cysteine, 100 mg kg‐1 day‐1), or L5+caffeine (400 μg/ml in free‐access drinking water) for 4 weeks (*n* = 5 per group). (a) SA‐β‐Gal staining in the thoracic aortic tissues of treated mice. (b) Immunofluorescence staining for p16^INK4a^ (red), TP53 (green), and LOX‐1 (red) in cross‐sections of thoracic aortic tissues from treated mice. (c) Immunofluorescence staining for γH2AX, a sensitive marker of DNA double‐strand breaks, in cross‐sections of thoracic aortic tissues from treated mice. Arrows indicate positive staining in the endothelium. Hoechst 33342 was used as nuclear marker. The merged image shows the nuclear localization of γH2AX foci. (d) Enlarged DAPI‐staining of representative images from treated mice. (e) Semi‐quantification of SA‐β‐Gal staining in thoracic aortic tissue samples from mice. Staining intensity was scored from 0 to 4, where 0 indicated no staining and 4 indicated fully stained. Scoring was performed by two independent, blinded researchers. The intensity scores were pooled, averaged, and analyzed using the Kruskal–Wallis test. Quantification of the percentage of cells that stained positive for (f) γH2AX or (g) senescence‐associated heterochromatin foci (SAHF). ***p < 0.001 vs. control; ^#^ *p* < 0.05, ^##^ *p* < 0.01, and ^###^ *p* < 0.001 vs. L5. L, vascular lumen

To examine the prosenescent and pro‐atherogenic properties of L5 in vivo, we administered L1 or L5 (2 mg kg^−1^ day^−1^) isolated from human plasma into C57B6/J mice via tail vein injection for 4 weeks and examined DNA damage and aortic endothelial senescence. No evidence of toxicity was associated with the injection of human L1 or L5 (Supporting Information Figure S1). In L5‐treated mice, the abundance of 3‐nitrotyrosine (a marker of cellular reactive oxygen species) in the aortic intima was higher than that in L1‐treated mice (Supporting Information Figures S2A and S3A). In addition, thoracic aortic tissues from mice treated with L5 showed intense blue deposits after senescence‐associated (SA)‐β‐Gal staining, whereas aortic tissues from mice treated with L1 showed staining comparable to that of normal saline‐treated control mice (Figure 1a,e). When we examined additional measures of cellular senescence, we found that the abundance of p16^INK4a^ and TP53 in the intimal layer of the aorta was higher in L5‐treated mice than in L1‐treated mice (Figure 1b; Supporting Information Figure S3B,C). These findings were in agreement with our SA‐β‐Gal staining results. When mice were co‐administered n‐acetyl cysteine (NAC, an anti‐oxidant) or caffeine (an ATM/ATR inhibitor), L5‐induced cellular oxidative stress and L5‐induced prosenescence were attenuated (Figure 1a,e; Supporting Information Figure S2A and S3A), suggesting that L5‐induced endothelial senescence may be dependent on intracellular reactive oxygen species (ROS) production or the DDR. In addition, L5 induced LOX‐1 expression in the thoracic aortic tissues of C57B6/J mice (Figure 1b; Supporting Information Figure S3D) but failed to induce endothelial senescence in LOX‐1^−/−^ mice. We previously showed that LOX‐1 serves as a receptor for L5 and mediates its entry into endothelial cells, subsequently inducing apoptosis (Li, Cao, Berndt, Funder, & Liu, 1999; Lu et al., 2008, 2009). Therefore, our results support that LOX‐1 mediates L5‐dependent endothelial senescence.

Because the activation of the DDR is a strong trigger for the cellular senescence response, we examined the presence of DNA damage in the thoracic aortic tissues of mice after L5 treatment. Immunofluorescence staining for γH2AX, the surrogate marker of the DDR (Rogakou, Pilch, Orr, Ivanova, & Bonner, 1998), and senescence‐associated heterochromatin foci showed that L5 treatment led to nuclear γH2AX deposition (Figure 1c,f) and senescence‐associated heterochromatin foci formation (Figure 1d,g) on the luminal side of the thoracic aorta.

In addition, L5‐induced nuclear γH2AX deposition and TP53 abundance were attenuated in the presence of NAC or caffeine or in LOX‐1^−/−^ mice (Figure 1b,c). These data suggest that electronegative L5 but not the relatively electroneutral L1 activates intracellular free‐radical production and the DDR in a LOX‐1‐dependent manner to induce endothelial senescence in vivo.

**Figure 2:**
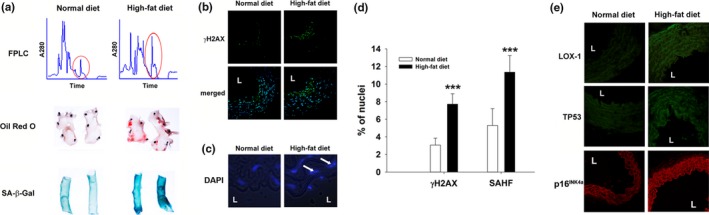
Prosenescent effect of endogenous L5 in vivo. Syrian hamsters were fed a high‐fat diet or a normal diet for 12 weeks (*n* = 6 per group). (a) Fast‐protein liquid chromatography (FPLC) results showing the quantification of LDL subfractions in hamsters fed a normal diet or a high‐fat diet. The peak corresponding to the L5 subfraction is circled red. Oil Red O staining (red) and SA‐β‐Gal (blue) in aortic tissues from both groups of hamsters. (b) Immunofluorescence staining for γH2AX in cross‐sections of thoracic aortic tissues from both groups of hamsters. Hoechst 33342 was used as a nuclear marker. The merged images show the nuclear localization of γH2AX. (c) Enlarged DAPI‐staining of representative images from both groups of hamsters. (d) Quantification of the percentage of cells that stained positive for γH2AX and senescence‐associated heterochromatin foci (SAHF). ***p < 0.001 vs. normal diet. (e) Immunofluorescence staining for LOX‐1 (green), TP53 (green), and p16^INK4a^ (red) in cross‐sections of thoracic aortic tissues from both groups of hamsters. L, vascular lumen

We next examined whether endogenous L5 is capable of triggering vascular endothelial senescence. Golden Syrian hamsters were placed on a normal or high‐fat diet for 3 months. LDL subfraction analysis by means of fast‐protein liquid chromatography showed that the concentration of L5 was higher in hamsters fed a high‐fat diet than that in hamsters fed a normal diet (Figure 2a). Histopathologic analysis of the thoracic aorta with Oil Red O and SA‐β‐Gal stains showed pronounced lipid accumulation and endothelial senescence, respectively, in the high‐fat diet group. Furthermore, γH2AX deposition (Figure 2b,d), senescence‐associated heterochromatin foci formation (Figure 2c,d), protein levels of LOX‐1, TP53, and p16^INK4a^ (Figure 2e; Supporting Information Figure S3E), and 3‐nitrotyrosine levels (Supporting Information Figures S2B and S3E) were more abundant in the high‐fat diet group than in the normal diet group. These data are consistent with our findings in mice treated with exogenous L5.

### 2.2 L5 induces the DDR and cellular senescence in cultured human aortic endothelial cells

**Figure 3:**
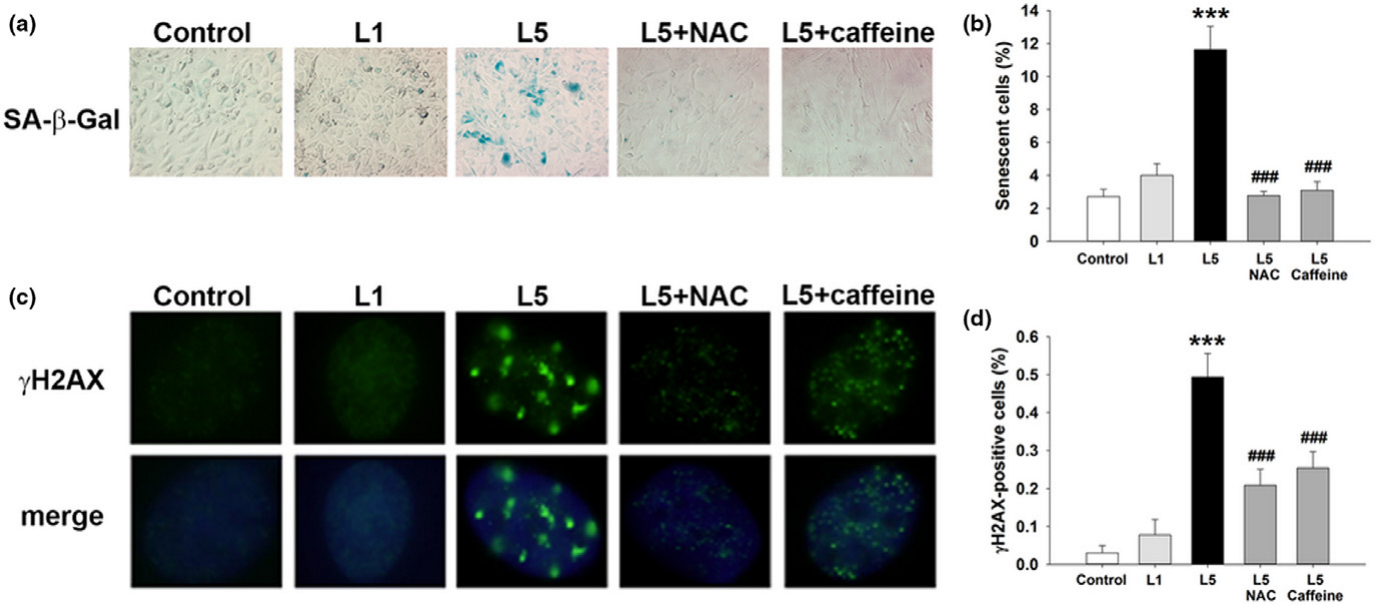
Prosenescent effect of L5 on human aortic endothelial cells (HAECs) in vitro. HAECs were treated with phosphate‐buffered saline (PBS) (control), L1 (30 μg/ml), L5 (30 μg/ml), L5 + NAC (5 mM), or L5 + caffeine (1 mM) for 72 hr (*n* = 4 independent experiments per treatment group). (a) SA‐β‐Gal staining in HAECs from each treatment group. Positively stained cells were quantified and are shown as percentages of the total number of cells in (b). (c) Immunofluorescence staining showing γH2AX foci in HAECs from each treatment group. Positively stained cells were quantified and are shown as percentages of the total number of cells in (d). DAPI (blue) counterstaining shows the nuclear localization of γH2AX. ***p < 0.01 vs. control; ^###^ *p* < 0.01 vs. L5

To further investigate the mechanisms underlying the association between L5 and vascular senescence, we conducted in vitro experiments with human aortic endothelial cells (HAECs). When HAECs were incubated with phosphate‐buffered saline, L1 (30 μg/ml), or a subapoptotic concentration of L5 (30 μg/ml) for 72 or 120 hr, cell growth inhibition was more prominent in L5‐treated cells than in L1‐treated cells (Supporting Information Figure S4). In addition, the number of SA‐β‐Gal‐stained cells (Figure 3a,b) and nuclear γH2AX foci (Figure 3c,d) was significantly higher in L5‐treated cells than in L1‐treated cells after 72 hr (*p* < 0.01 vs. control). Consistent with our findings in vivo, the L5‐induced increase in nuclear γH2AX foci and SA‐β‐Gal staining in HAECs was attenuated by NAC and caffeine (Figure 3a–d).

### 2.3 L5 induces mitochondrial superoxide production and enhanced mitochondrial respiration

**Figure 4:**
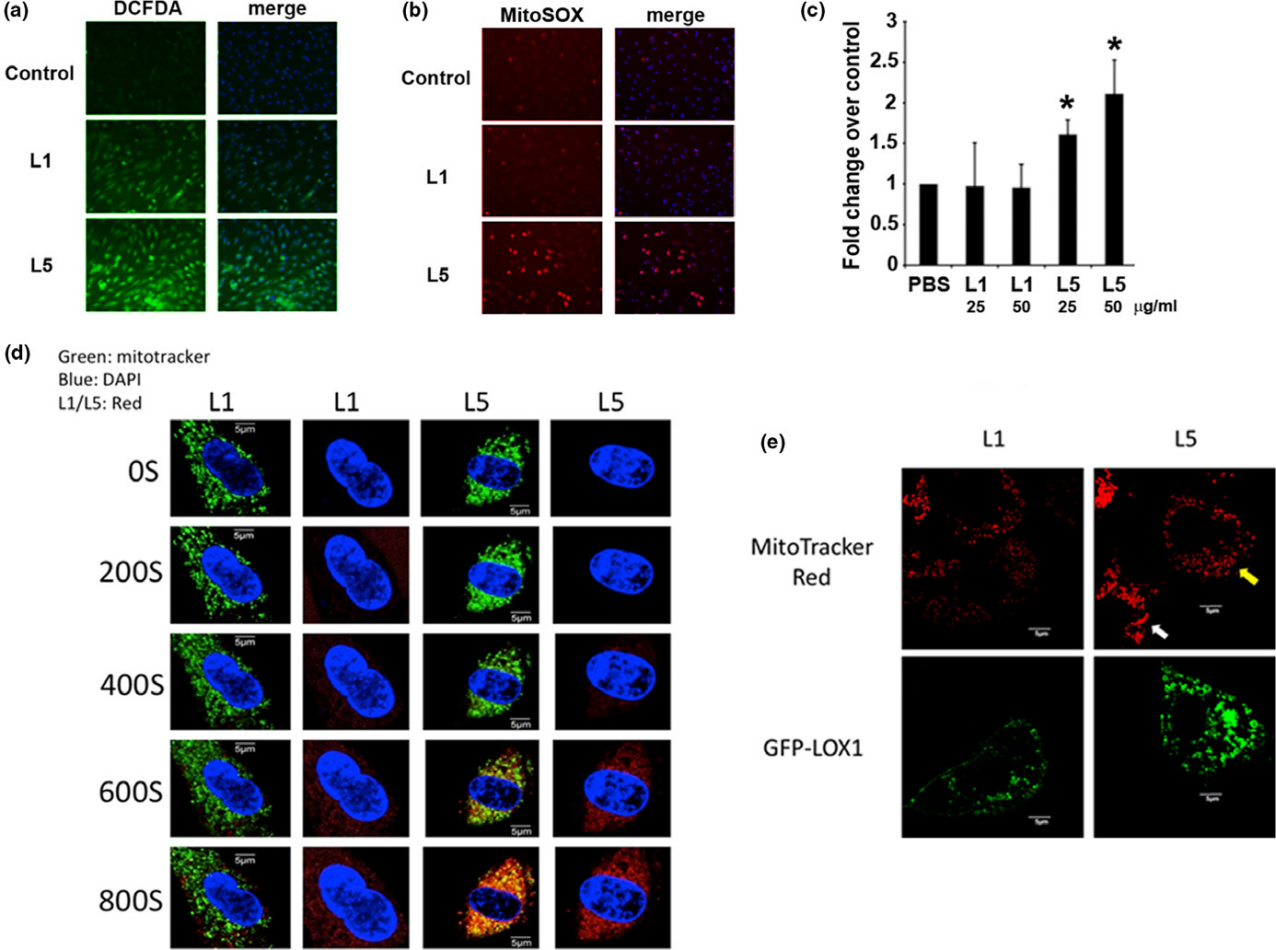
The effect of L5 on reactive oxygen species (ROS) formation and mitochondrial function in human aortic endothelial cells (HAECs). HAECs were exposed to L5 (50 μg/ml), L1 (50 μg/ml), or phosphate‐buffered saline (PBS) (control) for 24 hr (*n* = 4 per treatment group). Total intracellular ROS levels and mitochondrial superoxide production were measured by staining cells with (a) DCFDA and (b) MitoSOX Red, respectively. (c) Flow cytometry analysis of MitoSOX‐stained cells was used to confirm the L5 concentration‐dependent increase in mitochondrial superoxide production after L5 treatment. *p < 0.05 vs. control. (d) Live cell fluorescence microscopy analysis of the subcellular dynamics of L1 or L5. Mitochondria of HAECs cells were labeled with MitoTracker green, and L1 or L5 was labeled with CellTracker red. Yellow signals on the third column indicated the colocalization of lipoprotein to the mitochondria. (e) L5‐induced mitochondrial fission in LOX‐1‐expressing CHO cells. CHO cells were transiently transfected with green fluorescent protein (GFP)‐tagged LOX‐1 and treated with L1 or L5, and mitochondria were labeled with MitoTracker red. Cells were treated with L1 or L5 for 10 min, and mitochondrial morphology on confocal microscopy was recorded. Evidence of mitochondrial fission was observed only in LOX‐1‐expressing cells treated with L5 (yellow arrow). Cells that did not overexpress LOX‐1 retained an elongated and tubular mitochondrial morphology (white arrow)

Because NAC blocked L5‐induced DNA damage and cellular senescence, we examined whether oxidative stress is involved in L5‐induced endothelial senescence. Using DCFDA staining, we found that intracellular oxidative stress was increased in HAECs exposed to L5 (50 μg/ml) for 24 hr compared with control‐treated HAECs (Figure 4a). Because mitochondria are an important source of ROS and contribute to oxidative stress in cells under pathologic conditions, we also measured mitochondria‐derived ROS using MitoSOX stain. The results of fluorescence microscopy showed that, compared with L1 or saline treatment, L5 treatment led to increased mitochondrial superoxide production (Figure 4b). Moreover, quantitative analysis with flow cytometry indicated that the effects of L5 were dose dependent (Figure 4c). Detailed bioenergetic profiling with oxygen microfluorimetry showed that the preincubation of HAECs with L5 for 24 hr dose‐dependently increased basal respiration and maximal respiratory capacity (Supporting Information Figure S5 and Table S1), a phenomenon not seen within 3 hr after L5 treatment (data not shown). The amount of oxygen consumed for adenosine triphosphate production remained stationary after L5 treatment (Supporting Information Figure S5).

To examine whether L5 directly influences mitochondria, we traced the intracellular dynamics of L5 using live cell microscopy. Fluorescently labeled L5 but not L1 was rapidly taken up into HAECs and colocalized with mitochondria within 600 s (Figure 4d). We also examined the intracellular dynamics of L5 in Chinese hamster ovary (CHO) cells and found evidence of mitochondrial fission in L5‐treated CHO cells overexpressing LOX‐1 (Figure 4e) but not in CHO cells that did not overexpress LOX‐1. Chinese hamster ovary cells that did not overexpress LOX‐1 showed no uptake of L5, and their mitochondria retained a tubular appearance (Figure 4e). These results suggest that L5 treatment leads to rapid mitochondrial uptake and respiratory uncoupling and correlates with mitochondrial fission and subsequent ROS production. Mitochondrial ROS overload frequently leads to mitochondrial DNA damage and mitochondrial ROS depletion, which may explain the reduced appearance of PicoGreen, which labels mitochondrial nucleoids, and MitoTracker Red, which labels polarized mitochondria, in L5‐treated cells (Supporting Information Figure S6). In contrast, these phenomena were not seen in cells treated with relatively electroneutral L1.

### 2.4 L5 triggers endothelial senescence by activating the TP53‐dependent DDR and repressing human telomerase reverse transcriptase

**Figure 5:**
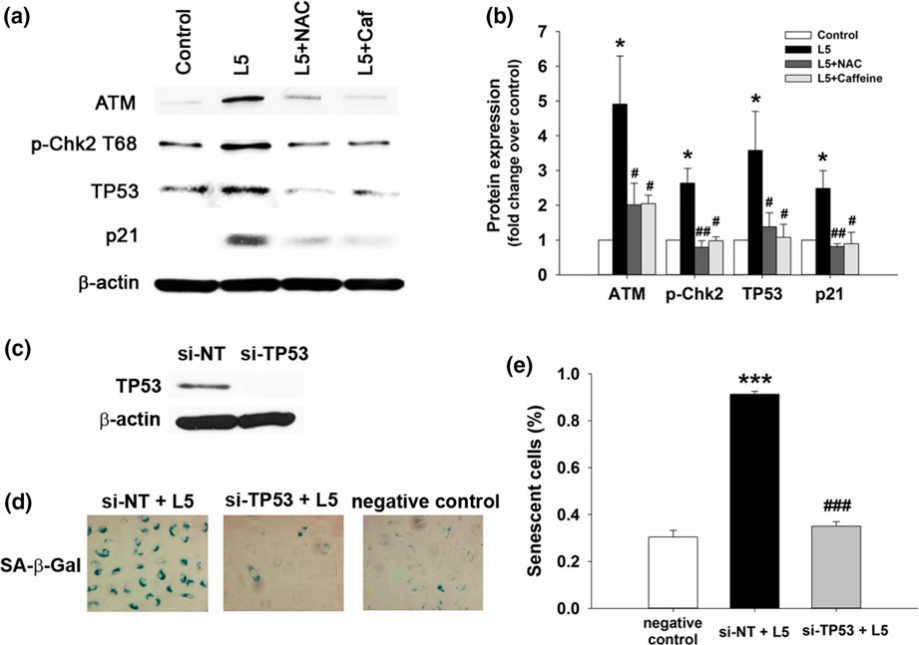
L5‐induced endothelial senescence in human aortic endothelial cells (HAECs) through the DNA damage response (DDR) pathway and TP53. (a) HAECs were treated with 30 μg/ml L5 or L1 for 5 days with or without pretreatment with 1 mM caffeine (Caf) or 5 mM NAC (*n* = 4 independent experiments per group). Western blot analysis showing ATM, phospho‐Chk2, TP53, and p21 protein expression. Western blot results are quantified in (b). *p < 0.05 vs. control; ^#^ *p* < 0.05 vs. L5; ^##^ *p* < 0.02 vs. L5. (c) Western blot analysis showing TP53 protein expression in HAECs transfected with nontargeting siRNA (si‐NT) or si‐TP53. (d) SA‐β‐Gal staining of HAECs transfected with si‐TP53 and treated with L5. Positively stained cells are quantified in (e). *n* = 4 independent experiments per group. ***p < 0.01 vs. negative control (untreated cells); ^###^ *p* < 0.01 vs. si‐NT+L5

To analyze the effects of L5 on the DDR, we treated cultured HAECs with a subapoptotic concentration of L5 (30 μg/ml) for 5 days and examined the activation of DDR pathways. Immunoblot analysis of total cell lysates showed that, consistent with the presence of nuclear DNA damage, L5 significantly increased the abundance of the DDR proteins ATM, p‐Chk2 T68, TP53, and p21 (Figure 5a,b; *p* < 0.05 vs. control). The cotreatment of cells with either NAC or caffeine inhibited the effects of L5 on DDR pathway activation. TP53 stabilization is critical for relaying the DDR and for executing the senescence response (Wang & Bennett, 2012; Zhan et al., 2010). We found that the small interfering RNA‐mediated silencing of TP53 in HAECs efficiently blocked the L5‐induced increase in SA‐β‐Gal staining and cellular senescence (Figure 5c–e).

**Figure 6:**
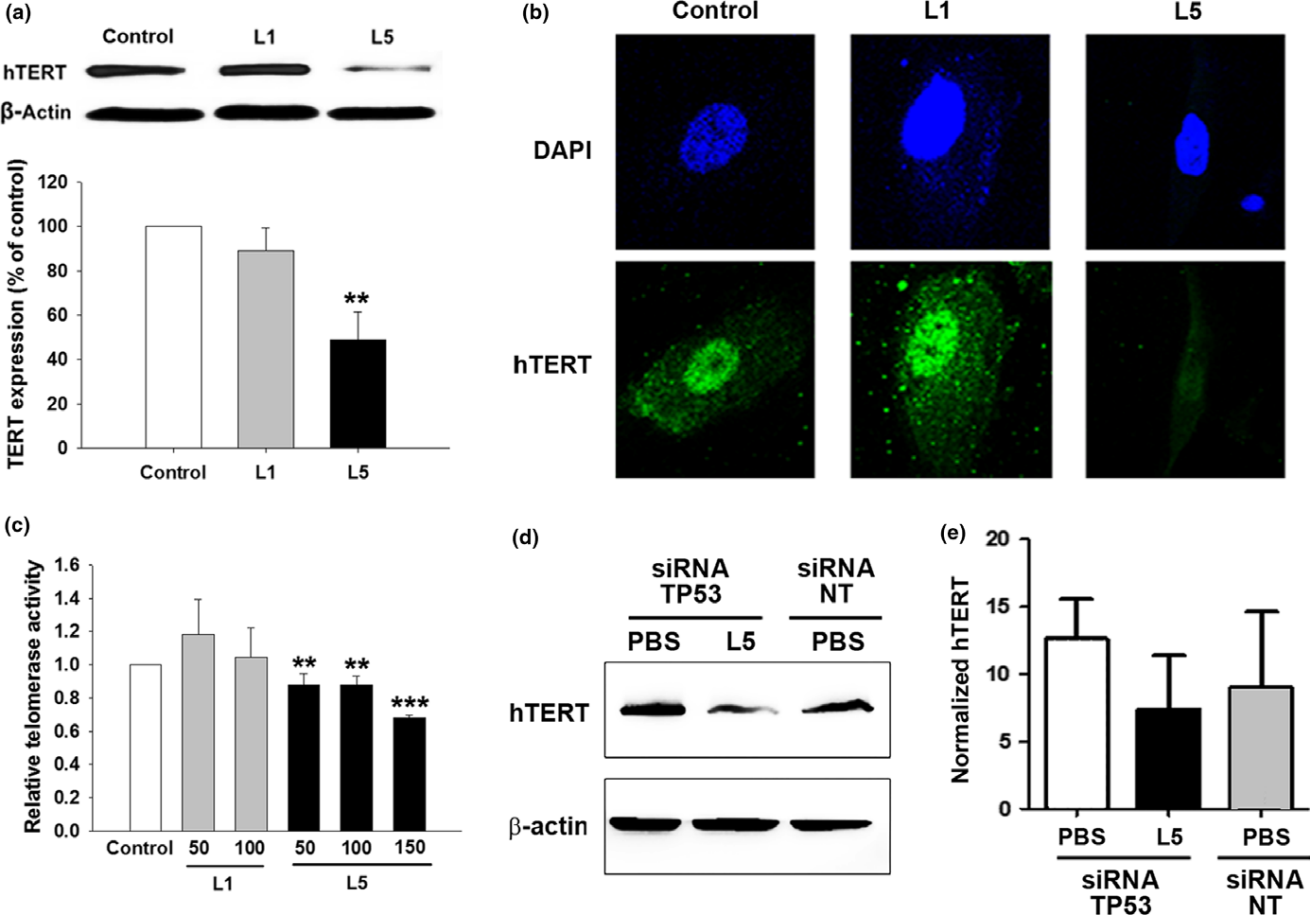
Inhibitory effects of L5 on telomerase expression and activity in human aortic endothelial cells (HAECs). HAECs were treated with phosphate‐buffered saline (PBS) (control), 50 μg/ml L1, or 50 μg/ml L5. (a) Representative western blot analysis showing hTERT protein expression in treated and control cells. Quantification of the results is shown (*n* = 3 independent experiments per group). **p < 0.02 vs. control. (b) Immunofluorescence staining of hTERT (green) in treated and control cells. DAPI was used as a nuclear counterstain (blue). (c) Telomerase activity of cells treated with PBS, 50 or 100 μg/ml L1, or 50 or 100 μg/ml L5. *n* = 3 independent experiments per group. **p < 0.02 and ***p < 0.01 vs. control. (d) Representative western blot analysis after experiments with siRNA against TP53 (siRNA TP53) and control siRNA (nontargeting siRNA, siRNA NT). TP53 is required for the L5‐induced downregulation of hTERT expression in HAECs. (e) Quantification of the western blot results shown in (d) from three independent experiments. *p < 0.05 vs. nontargeting siRNA (siRNA NT)

Repressed telomerase activity, which is known to follow the DDR and TP53 stabilization (Li et al., 1999), is associated with shortened telomere length and therefore activates the senescence response. Because we found that L5 treatment led to TP53 stabilization, we examined whether telomerase expression and activity are also repressed. Immunoblot (Figure 6a) and immunofluorescence (Figure 6b) analyses of HAECs showed that treatment with 50 μg/ml L5 but not 50 μg/ml L1 for 24 hr significantly decreased the expression of human telomerase reverse transcriptase (hTERT), a critical protein component of telomerase (*p* < 0.02 vs. control). The treatment of HAECs with L5 not only reduced hTERT protein expression, but also significantly inhibited telomerase activity in a dose‐dependent manner (*p* < 0.02 or *p* < 0.01 vs. control; Figure 6c). Moreover, L5‐induced hTERT downregulation was attenuated after the small interfering RNA‐mediated knockdown of TP53 (Figure 6d,e).

## 3 DISCUSSION

In the present study, we have shown for the first time to our knowledge that human electronegative L5 LDL induces endothelial cell senescence, both in vivo and in vitro, whereas the relatively electroneutral L1 LDL, does not. In addition, we showed in hamsters that a high‐fat diet led to an elevated serum level of L5 and aortic endothelial senescence. These results provide evidence of a causal role of L5 in endothelial senescence and support our previous finding that L5 has a pathologic role in atherosclerotic formation and progression. Moreover, we showed in cultured HAECs that L5 rapidly localizes to mitochondria after cellular uptake and that L5‐induced endothelial senescence is mediated through mitochondrial ROS formation, the subsequent activation of the DDR, and decreased telomerase activity.

In the past, in individuals with ischemic heart disease, it was shown that coronary endothelial cells from plaque are senescent (Minamino et al., 2002). Vascular senescence may contribute to adverse vascular remodeling before atherosclerosis development (Wang & Bennett, 2012). In the past, we showed that L5 can induce the apoptosis of endothelial cells and cardiomyocytes (Lee et al., 2012; Lu et al., 2008). In the present study, we treated HAECs with a lower concentration of L5 than was previously used, and cellular senescence but not apoptosis was observed. Therefore, these findings imply that cellular senescence may mediate endothelial dysfunction and atherogenesis after chronic exposure to a sublethal level of L5. In other studies, we have shown that the concentration of L5 is less than 10 μg/ml in healthy individuals and is 100 μg/ml in patients with acute myocardial infarction (Chan et al., 2013), 20 μg/ml in patients with chronic kidney disease (Chang et al., 2013, 2016), and 100 μg/ml in patients with hypercholesterolemia (Chen et al., 2003). These estimations indicate that the concentration of L5 chosen for our in vitro experiments is the same order of magnitude as the concentration detected in humans, suggesting that our findings may be clinically relevant. In addition, we previously found that the percentage of L5 in total LDL from patients with myocardial infarction was around 15% (Chan et al., 2013), which is equivalent to an L5 plasma level of approximately 15 mg/dl. If the effects of redistribution and plasma protein binding are omitted, the dosage of L5 used to inject the mice (2 mg kg^−1^ day^−1^) would presumably reach a plasma concentration of 15 mg/dl.

It is well known that ROS‐induced oxidative stress and the DDR have critical roles in vascular aging and atherosclerosis (Finkel & Holbrook, 2000; Fleenor, Seals, Zigler, & Sindler, 2012; Wang & Bennett, 2012). Oxidative stress originates primarily from the mitochondria (Minamino & Komuro, 2007) and diffuses to the nucleus to activate the DDR, leading to cellular senescence (Mahmoudi, Mercer, & Bennett, 2006). This has been reported for human umbilical vein endothelial cells (Zhan et al., 2010), plaque‐derived vascular smooth muscle cells (Bennett, Evan, & Schwartz, 1995), and ApoE^−/−^ mice (Mercer et al., 2012). In this study, we showed that a sublethal concentration of L5 stimulated mitochondrial ROS formation in HAECs, whereas L1 treatment had only a modest effect on intracellular oxidative stress and did not lead to increased ROS in the mitochondria or induce the senescence response. Thus, it appears that the induction of mitochondrial ROS by L5 is relevant to its pathogenic action. We also found that the L5‐induced formation of mitochondrial ROS in HAECs occurred upstream of the activation of ATM, which in turn occurred upstream of the development of DNA damage foci and the induction of proteins in the DDR cascade, including Chk2 and TP53. TP53 activation may downregulate telomerase activity and hTERT abundance in cancer cells (Li et al., 1999; Xu et al., 2000). We found that such phenomena also occurred in HAECs and that TP53 expression was required to mediate hTERT downregulation after L5 treatment. Furthermore, we found that by blocking the DDR with NAC, caffeine, or TP53 silencing, the L5‐induced senescence of HAECs was inhibited. We believe our data provide sufficient evidence to support the notion that a sublethal concentration of electronegative L5 causes endothelial cell senescence through the DDR pathway and telomerase dysfunction.

In this study, a key role for L5 in endothelial senescence is reinforced by evidence from two experimental animal models: golden Syrian hamsters with endogenously elevated L5 levels and C57B/6 mice intravenously injected with human L5. The likelihood that our findings in mice injected with human L5 are confounded by a xeno‐immune response to human lipoproteins is minimal for the following reasons. First, similar findings were observed in hamsters with endogenously elevated levels of L5. Second, human‐derived L1 did not induce DNA damage or prosenescent activity. Third, in mice genetically deficient for the LOX‐1 receptor that mediates the uptake of L5 but not L1, L5‐induced DNA damage and endothelial senescence were blocked. It is also noteworthy that we have reported that L5 serum levels are elevated in metabolic syndrome patients and positively correlated with the number of metabolic risk factors (Hsu et al., 2014). It would be of interest to follow up these patients and test whether serum levels of L5 independently predict atherosclerotic vascular events.

On the basis of our findings, we have devised a working model for the mechanism of L5 in endothelial cell senescence (Supporting Information Figure S7). The exposure of endothelial cells to L5 and the uptake of L5 via LOX‐1 result in accelerated mitochondrial respiration and mitochondria‐derived ROS. Presumably, this in turn damages nuclear DNA and activates the DDR cascade for the stabilization of TP53. As a result, TP53 upregulates p21 expression and represses telomerase activity to induce the senescence phenotype. Our findings show a critical role for L5 in vascular senescence and suggest that L5‐induced endothelial senescence may be a therapeutic target in the management of atherosclerotic vascular diseases.

## 4 EXPERIMENTAL PROCEDURES

### 4.1 Animals

All animal experiments were approved by the China Medical University Institutional Animal Care and Use Committee in accordance with the National Institute of Health's Guide for the Care and Use of Laboratory Animals. For experiments examining the effects of exogenous L5, thirty 8‐week‐old male C57B6/J mice (BioLASCO Taiwan Co., Ltd., Taipei, Taiwan) and six 8‐week‐old male lectin‐like oxidized LDL receptor‐1 (LOX‐1) knockout mice (LOX‐1^−/−^) weighing 20–25 g were used. Our laboratory routinely backcrosses LOX‐1^−/−^ mice with wild‐type C57B6/J mice to minimize differences in genetic background. Mice were randomly designated to receive one of the following treatments (*n* = 6 per group): saline, L1, L5, L5 + NAC, or L5 + caffeine. LDL was injected via the tail vein once a day for 4 weeks. To examine the effects of endogenous L5, twenty 8‐week‐old male golden Syrian hamsters (National Laboratory Animal Center, Taipei, Taiwan) weighing 100–120 g were fed with a normal diet or a high‐fat diet (TestDiet 58Y1) for 3 months. Throughout the study, we used L1, which is normal LDL, as a control to rule out the possibility of a xeno‐antigen‐induced immune response.

### 4.2 LDL isolation and separation

This study was approved by the institutional review board of China Medical University Hospital in Taiwan. Plasma LDL was isolated from patients with metabolic syndrome using sequential potassium bromide density‐gradient ultra‐centrifugation (density = 1.019–1.063 g/ml) and supplemented with protease inhibitor cocktail, 1% penicillin/streptomycin/neomycin mixture, and 0.5 mM EDTA. Isolated LDL was separated into five subfractions (L1–L5) using an anion‐exchange fast‐protein liquid chromatography system (GE Healthcare, Princeton, NJ, USA), as described previously (Chen et al., 2003). The bioactivity of each batch of L5 was determined by examining its ability to induce endothelial cell apoptosis in 24 hr (Chen et al., 2003). The bioactivity of L5 was then used to normalize the concentration of L5 used in subsequent animal and cell experiments.

For LDL isolated from hamsters, plasma was prepared from 5 mL of whole blood anticoagulated with EDTA and was subjected to the same process described above for the separation of LDL subfractions.

### 4.3 SA‐β‐Gal staining

SA‐β‐Gal staining of cultured HAECs and mouse and hamster aortas was performed using an SA‐β‐Gal Staining Kit (Cell Signaling Technology, Danvers, MA, USA) according to the manufacturer's instructions. Senescent cells were identified as blue‐stained cells using microscopy. The semi‐quantification of SA‐β‐Gal staining is described in the Supplemental Methods.

### 4.4 Oil Red O staining

Hamsters were anesthetized by 5% isoflurane inhalation and euthanized by means of cervical dislocation, followed by removal of the descending thoracic aorta. To visualize atherosclerotic plaques, aortic arches were fixed with 4% paraformaldehyde and stained with Oil Red O (Sigma, St. Louis, MO, USA) for 1 hr.

### 4.5 Cell treatment

To evaluate the effect of L5 on cellular senescence, HAECs were incubated with a subapoptotic concentration of L5 (30 μg/ml), L1 (30 μg/ml), or phosphate‐buffered saline (lipoprotein‐free control) continuously for 5 days. Caffeine (1 mM) or NAC (5 mM) was added to cells incubated with L5 to evaluate their effects on L5‐induced senescence (Lu et al., 2009).

### 4.6 Measurement of intracellular ROS and mitochondrial superoxide

To examine the intracellular ROS levels and mitochondrial superoxide production, LDL‐treated HAECs were treated with carboxy‐H2DCFDA and MitoSOX solutions (Invitrogen, Grand Island, NY, USA) and analyzed with either fluorescence microscopy or flow cytometry.

### 4.7 Analysis of subcellular dynamics and mitochondrial fission

The subcellular dynamics of L1 or L5 were analyzed using live cell fluorescence microscopy. Mitochondria of HAECs cells were labeled with MitoTracker green (Invitrogen), and L1 or L5 was labeled with CellTracker red (Invitrogen). The dynamics of L1 and L5 in HAECs were captured every 200 s for 800 s. L5‐induced mitochondrial fission was examined in LOX‐1‐expressing Chinese hamster ovary (CHO) cells. Chinese hamster ovary cells were transiently transfected with green fluorescent protein (GFP)‐tagged LOX‐1 and treated with L1 or L5. Mitochondria were labeled with MitoTracker red (Invitrogen), and mitochondrial morphology was examined using confocal microscopy.

### 4.8 Mitochondrial DNA staining

Mitochondrial DNA staining in HAECs was performed by adding PicoGreen solution (Invitrogen) directly into cell culture medium for 1 hr. Cells were counterstained with the mitochondrion‐selective dye MitoTracker Red FM (Invitrogen), rinsed, and directly visualized using epifluorescence microscopy.

### 4.9 Immunofluorescence analysis

Slides containing serial cross‐sections of thoracic descending aortic tissue or HAECs were immunostained with antiphosphorylated histone H2AX (anti‐γH2AX) antibody (Cell Signaling Technology) and then with Alexa‐488 conjugated secondary antibody. Hoechst 33342 or DAPI (4′,6‐diamidino‐2‐phenylindole) was used to stain nucleic acids and to assess the formation of senescence‐associated heterochromatin foci. Anti–LOX‐1 (Santa Cruz Biotechnology, Dallas, TX), anti‐TP53 (Cell Signaling Technology), anti‐p16^INK4a^ (Abcam Inc., Cambridge, MA), and anti‐3‐nitrotyrosine (EMD Millipore, Darmstadt, Germany) antibodies were used to determine in situ the abundance of LOX‐1, TP53, p16^INK4a^, and 3‐nitrotyrosine in aortic tissue samples. Anti‐hTERT monoclonal antibody (EMD Millipore, Billerica, MA, USA) was used to detect hTERT expression in HAECs. (Klokov, MacPhail, Banath, Byrne, & Olive, 2006). The semi‐quantification of immunofluorescence staining is described in the Supplemental Methods.

### 4.10 Determination of telomerase activity

Telomerase activity was measured using a telomerase repeat amplification assay (TRAPeze Telomerase Detection Kit, Chemicon, Temecula, CA, USA) according to the manufacturer's instructions. Human aortic endothelial cells were lysed with CHAPS buffer, and lysate from 5,000 cells was used for each reaction. The resultant amplicon abundance was measured using quantitative PCR.

### 4.11 Statistical analysis

Continuous data are expressed as the mean ± standard error of the mean for normally distributed variables. Differences between two groups were analyzed using a Student *t* test. For the semi‐quantification of SA‐β‐Gal and immunofluorescence staining, the intensity scores were analyzed using the Kruskal–Wallis test. A probability (*p*)‐value <0.05 was considered statistically significant.

## CONFLICT OF INTEREST

The authors have no conflict of interest to declare.

## AUTHOR CONTRIBUTIONS

YCW initiated the research, designed research studies, conducted experiments, acquired data, analyzed data, and wrote the manuscript. ASL designed research studies, conducted experiments, acquired data, analyzed data, and wrote the manuscript. LSL initiated the research, designed research studies, conducted experiments, acquired data, analyzed data, and wrote the manuscript. LYK conducted experiments, acquired data, and analyzed data. WYC, JWD, ZPC, CSC, HCC, TYK, MHT, and WLH conducted experiments and acquired data. JL conducted experiments, acquired data, and analyzed data. RAFD designed research studies. TS designed research studies and provided reagents. KCC designed research studies, analyzed data, and wrote the manuscript. CHC initiated the research, designed research studies, analyzed data, and wrote the manuscript. All authors approved the final version of the manuscript.
