# Supplementary material for: Human electronegative LDL induces mitochondrial dysfunction and premature senescence of vascular cells in vivo
Source: Aging Cell. 2018 Jun 19;17(4):e12792. doi: 10.1111/acel.12792 (PMC6052487; doi:10.1111/acel.12792)

**Supporting Information**

**Supplemental experimental procedures**

**Treatment of mice**

For experiments examining the effects of exogenous L5, thirty 8-week-old male C57B6/J mice (BioLASCO, Taiwan Co., Ltd.) weighing 20 to 25g were randomly designated to receive one of the following treatments (n=6 per group): saline, L1, L5, L5+n-acetyl cysteine (NAC), or L5+caffeine. Once a day for 4 weeks, 100 μl normal saline or an equal volume of 2 mg/kg L1 or L5 was intravenously injected through the tail vein of mice. For NAC, 100 mg/kg was intraperitoneally injected 30 min before L5 injection. For caffeine, 400 μg/mL was administered in free-access drinking water. Similarly, six 8-week-old male lectin-like oxidized LDL receptor-1 (LOX-1) knockout mice (LOX-1^-/-^) from the laboratory of Tatsuya Sawamura were injected with L5 (2 mg/kg/day) for 4 weeks.

**Cell culture**

For cell studies, primary human aortic endothelial cells (HAECs) were cultured in endothelial cell growth medium 2 (EGM2; Lonza, Basel, Switzerland) as previously described (Jo-Watanabe *et al.,* 2014). Before cellular assays, HAECs were cultured in 12-well plates until the cells reached 80% confluency.

**Senescence-associated β-galactosidase (SA-β-Gal) staining**

Mice and hamsters were anesthetized by 5% isoflurane inhalation and euthanized by means of cervical dislocation, followed by removal of the descending thoracic aorta. SA-β-Gal staining of mouse and hamster aortas and cultured HAECs was performed by using a Senescence-associated β-Galactosidase Staining Kit (Cell Signaling Technology, Danvers, MA) according to the manufacturer’s instructions. Images were acquired with a Nikon D300s digital camera, and senescent cells were identified as those marked with a blue color produced by the enzymatic reaction (Burnley *et al.,* 2013). More than 30,000 cells total were counted in 9 randomly selected fields to determine the percentage of SA-β-Gal–positive cells.

For the semi-quantification of SA-β-Gal staining, images were analyzed from 8 different samples per group. The staining intensity was scored from 0-4, where 0 indicated no staining and 4 indicated fully stained. Scoring was performed by 2 independent, blinded researchers. The intensity scores were pooled, averaged, and analyzed by using the Kruskal-Wallis test.

**Immunofluorescence analysis**

Slides containing serial cross-sections of thoracic descending aortic tissues were immunostained with anti-phosphorylated histone H2AX (anti-γH2AX) antibody (Cell Signaling Technology) and with Hoechst 33342 as a nuclear counterstain. Anti–LOX-1 (Santa Cruz Biotechnology, Dallas, TX), anti-TP53 (Cell Signaling Technology), anti-3-nitrotyrosine (EMD Millipore, Darmstadt, Germany), and anti-p16 ^INK4a^ antibodies were used to determine *in situ* LOX-1, TP53, 3-nitrotyrosine, and p16^INK4a^ expression in aortic tissue samples.

To detect γH2AX in HAECs, cells were washed with cytoskeletal buffer before fixation. Cells were blocked and incubated first with anti-γH2AX monoclonal antibody (Cell Signaling Technology) and then with Alexa-488 conjugated secondary antibody. Cells were also stained with DAPI as a nuclear counterstain. Cells with ≥3 foci were considered positive for γH2AX foci. γH2AX foci were counted in more than 50 cells per field in 9 randomly selected fields per group. Similar procedures (with the exception of pretreatment with cytoskeletal buffer) were used to detect human telomerase reverse transcriptase (hTERT) expression with anti-hTERT monoclonal antibody (EMD Millipore, Billerica, MA, USA).

For the semi-quantification of LOX-1, TP53, p16^INK4a^, and 3-nitrotyrosine immunofluorescence staining, images were analyzed from 4 different samples per group (2 images per sample). We segmented signals from the inner fourth portion of the vascular ring to minimize confounding from vascular smooth muscle cells. The staining intensity was scored from 0-4, where 0 indicated no staining and 4 indicated fully stained. Scoring was performed by 2 independent, blinded researchers. The intensity scores were pooled, averaged, and analyzed by using the Kruskal-Wallis test.

**Measurement of mitochondrial oxygen consumption rate**

Mitochondrial oxygen consumption was measured by means of microfluorimetry according to an established protocol (Seahorse XF96 Analyzer, Seahorse Bioscience, North Billerica, MA, USA). Briefly, HAECs were incubated with L1 (25 or 50 μg/ml), L5 (25 or 50 μg/ml), or phosphate-buffered saline for 24 hours. A sensor cartridge was applied to the cells, and the oxygen consumption rate was continuously monitored for 3 hours during the serial administration of oligomycin (1 μg/ml) and carbonyl cyanide-4-(trifluoromethoxy) phenylhydrazone (FCCP; 0.5 μM) or oligomycin and antimycin A (2 μg/ml). Afterwards, the cells were lifted with trypsin and were enumerated with a capacitance-based cell counter for well-to-well oxygen consumption rate normalization (Multisizer 3, Beckman Coulter, Brea, CA, USA). For each group, 4 to 8 samples were analyzed.

Basal mitochondrial respiration was defined as normalized oxygen consumption rate (basal) minus normalized oxygen consumption rate (nadir post rotenone), and maximal mitochondrial respiration was defined as normalized oxygen consumption rate (peak post FCCP) minus normalized oxygen consumption rate (nadir post rotenone).

**Western blot analysis**

HAECs were lysed in radioimmunoprecipitation assay buffer with protease inhibitor cocktail. Denatured whole cell lysates were separated with sodium dodecyl sulfate polyacrylamide gel electrophoresis and were blotted onto PVDF membrane. Primary antibodies included anti-ATM (Abcam, Cambridge, MA, USA), anti-pChk2 T68 (R&D Systems, Minneapolis, MN, USA), anti-TP53 (Cell Signaling Technology), anti-p21 (Abcam), anti-hTERT (Millipore), and anti–β-actin (Sigma-Aldrich Co. LLC, St. Louis, MO, USA).

**Supplemental figure legends**

**Figure S1** Comparison of serum levels of creatinine (A) and blood urea nitrogen (B) levels between all groups of mice.

**Figure S2** Immunofluorescence staining for nitrotyrosine (red) in cross-sections of thoracic aortic tissues. (A) Aortas from mice injected with 2 mg/kg human L1 or L5 and treated with the indicated drug. Normal saline was used as a control. Wild-type mice were used unless otherwise specified. NAC, n-acetyl cysteine. (B) Aortas from hamsters fed a normal chow or high-fat diet. n=4 per group.

**Figure S3** Semi-quantification of 3-nitrotyrosine immunofluorescence, p16^INK4a^, TP53, and LOX-1 staining in thoracic aortic tissue samples from mice (A-D) and hamsters (E). Staining intensity was scored from 0-4, where 0 indicated no staining and 4 indicated fully stained. Scoring was performed by 2 independent, blinded researchers. The intensity scores were pooled, averaged, and analyzed by using the Kruskal-Wallis test. ***P*<0.01 and ****P*<0.001 vs. control or normal diet; ###*P*<0.001 vs. L5.

**Figure S4** Growth inhibition effects of L5 on cultured human aortic endothelial cells (HAECs) *in vitro*. HAECs were treated with phosphate-buffered saline (PBS), L1 (30 μg/mL), or L5 (30 μg/mL) for 24 (Day 1), 72 (Day 3), or 120 hours (Day 5) (n=4 independent experiments per treatment group). Cells were counted by using a Coulter counter. After 72 and 120 hours of incubation, the effects of L5 on cellular growth inhibition were more prominent than those of L1. **P*<0.05 and ***P*<0.01 vs. PBS; #*P*<0.05 vs. L1. *P*-values were determined by using a Student *t*-test.

**Figure S5** Oxygen microfluorimetry tracings and mitochondrial respiratory profile.. Human aortic endothelial cells (HAECs) were pretreated with 25 or 50 μg/mL of L5 or L1 or phosphate-buffered saline (PBS; negative control) for 24 hours before the start of the experiment. The oxygen consumption rates (OCRs) were analyzed online every 8 minutes. In (A) and (B), the injection of oligomycin (1 μg/mL) was indicated at point A, and the injection of carbonyl cyanide-4-(trifluoromethoxy) phenylhydrazone (FCCP; 0.5 μM) was indicated at point D. In (C) and (D), the injection of FCCP was indicated at point A, and the injection of antimycin A (2 μg/mL) was indicated at point D. Baseline and post-FCCP OCRs were higher in the L5 (50 μg/mL)–pretreated groups than in the PBS-treated controls. (E-G) Mitochondrial respiratory profile measured by using oxygen microfluorimetry. The oxygen consumption rate is expressed in pmol/min. Basal respiration refers to mitochondria-derived oxygen consumption; maximal respiration refers to maximal mitochondrial oxygen consumption after FCCP uncoupling; and adenosine triphosphate turnover refers to oxygen consumed for adenosine triphosphate production, as measured by using the oligomycin-sensitive oxygen consumption rate. L5 but not L1 dose-dependently increased basal and maximal mitochondrial respiratory capacity. Each measurement was performed in 6 replicates, and 3 independent experiments were performed.

**Figure S6** Analysis of mitochondrial DNA with fluorescent dyes in human aortic endothelial cells (HAECs) treated with phosphate-buffered saline (PBS), L1, or L5. Nuclear DNA and mitochondrial nucleoids were detected with PicoGreen (green). Mitochondria were labeled with the mitochondrial marker MitoTracker Red (red). The co-localization of PicoGreen with MitoTracker is shown in yellow, which indicates DNA mass in the mitochondria (mitochondrial nucleoids).

**Figure S7** Schematic mechanism of L5-induced endothelial senescence in humans. Endogenous L5, the most electronegative LDL subfraction, binds to LOX-1 and induces mitochondrial uncoupling for reactive oxygen species (ROS) production. These ROS cause double strand breaks in genomic DNA and activate the DNA damage response (DDR) cascade involving ATM, Chk2, and TP53. TP53 accumulation leads to the upregulation of p21 expression and the downregulation of telomerase protein expression and activity, which all contribute to the induction of cellular senescence.

**Table S1** Bioenergetic profile of human aortic endothelial cells (HAECs) in response to L1 and L5 treatment

|  | Basal respiration (pmol O_2_/min) | Maximal respiration (pmol O_2_/min) | Oxygen consumed for ATP turnover  (pmol O_2_/min) | Leak respiration (pmol O_2_/min) | Non-mitochondrial respiration  (pmol O_2_/min) |  | Spare respiratory capacity  (pmol O_2_/min) |
| --- | --- | --- | --- | --- | --- | --- | --- |
| PBS | 73 ± 4 | 225 ± 17 | 81 ± 4 | –8 ± 4 | 55 ± 2 |  | 47 ± 13 |
| L5 (25 μg/ml) | 76 ± 4 | 246 ± 17 | 75 ± 5 | 1 ± 3 | 53 ± 1 |  | 63 ± 12 |
| L5 (50 μg/ml) | 85 ± 4* | 265 ± 9* | 77 ± 9 | 8 ± 7* | 56 ± 1 |  | 60 ± 13 |
| L1 (25 μg/ml) | 69 ± 7 | 229 ± 15 | 69 ± 8 | 0 ± 5 | 50 ± 2 |  | 61 ± 11 |
| L1 (50 μg/ml) | 72 ± 4 | 212 ± 6 | 63 ± 7 | 9 ± 7 | 51 ± 2 |  | 70 ± 11 |

**P*<0.05 vs. PBS-treated group (determined by using a Student *t*-test).

Basal respiration, maximal respiration, oxygen consumed for adenosine triphosphate turnover, leak respiration, non-mitochondrial respiration, and spare respiratory capacity were derived from oxygen microfluorimetry tracings in response to the sequential application of oligomycin, carbonyl cyanide-4-(trifluoromethoxy) phenylhydrazone (FCCP), and antimycin A, as shown in Fig. S1. Profiles were derived from a representative experiment. Similar trends were observed in 2 additional independent experiments. PBS, phosphate-buffered saline.

**References**

Burnley P, Rahman M, Wang H, Zhang Z, Sun X, Zhuge Q, Su DM (2013) Role of the p63-FoxN1 regulatory axis in thymic epithelial cell homeostasis during aging. *Cell Death Dis*. **4**, e932.

Jo-Watanabe A, Ohse T, Nishimatsu H, Takahashi M, Ikeda Y, Wada T, Shirakawa JI, Nagai R, Miyata T, Nagano T, Hirata Y, Inagi R, Nangaku M (2014) Glyoxalase I reduces glycative and oxidative stress and prevents age-related endothelial dysfunction through modulation of endothelial nitric oxide synthase phosphorylation. *Aging Cell*. **13**, 519–528.

Fig. S1


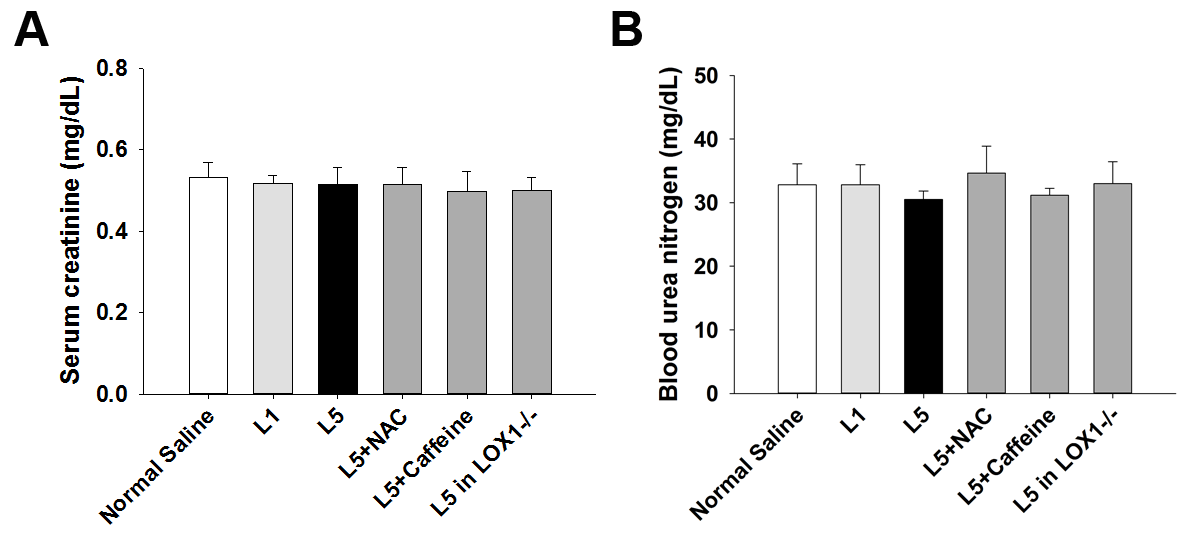


Fig. S2


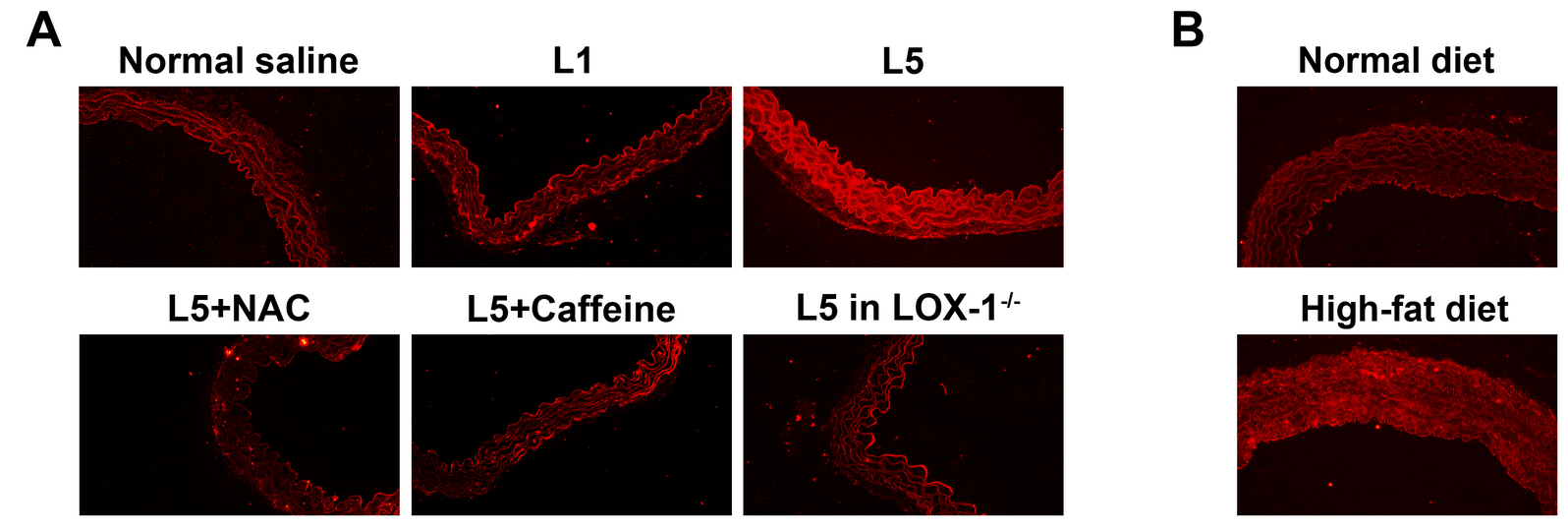


Fig. S3


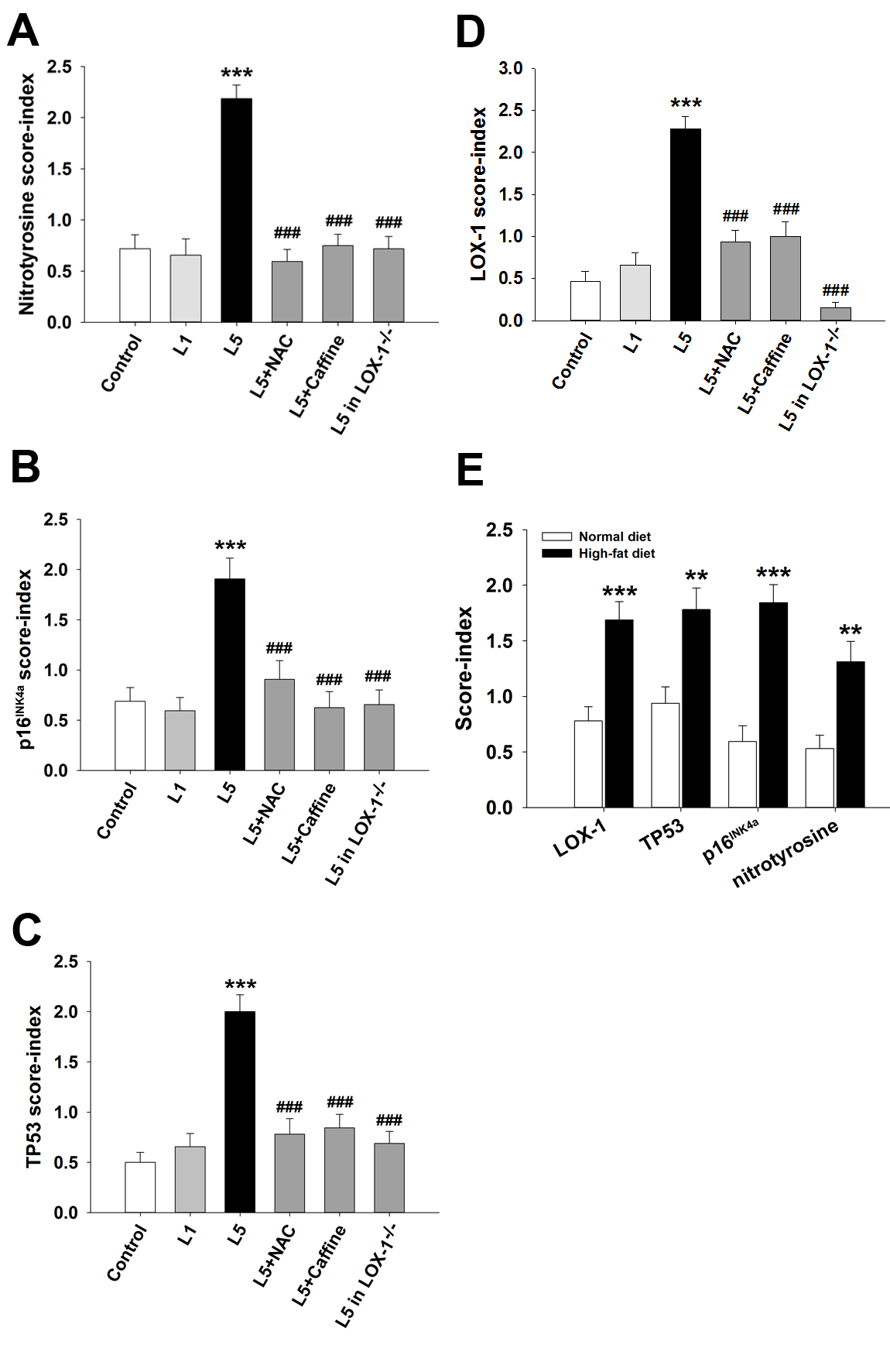


Fig. S4





Fig. S5


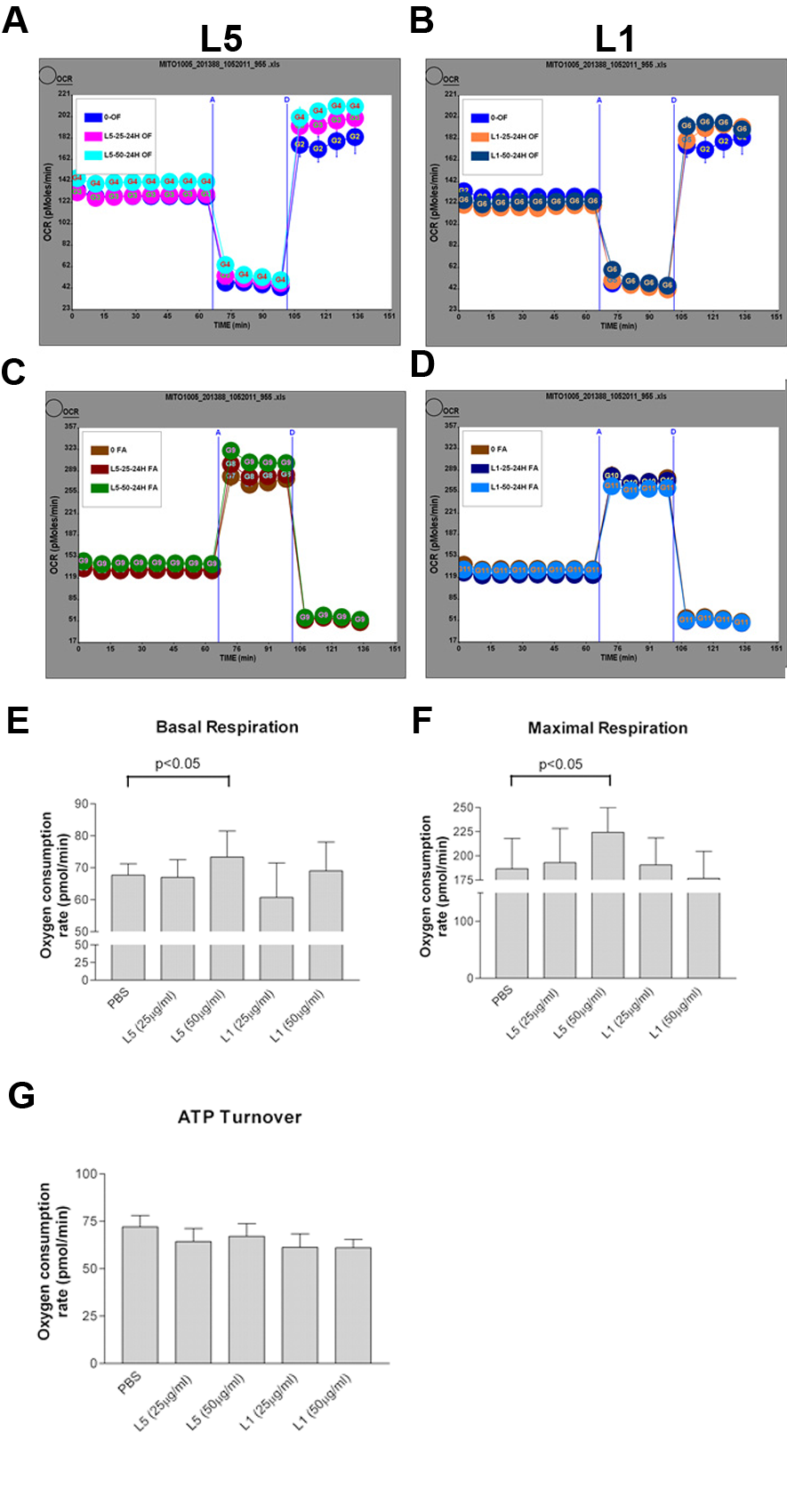


Fig. S6


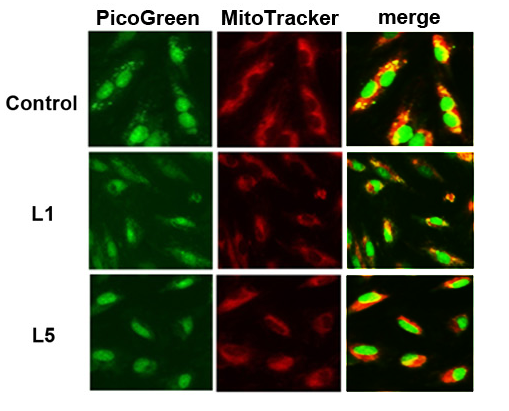


Fig. S7


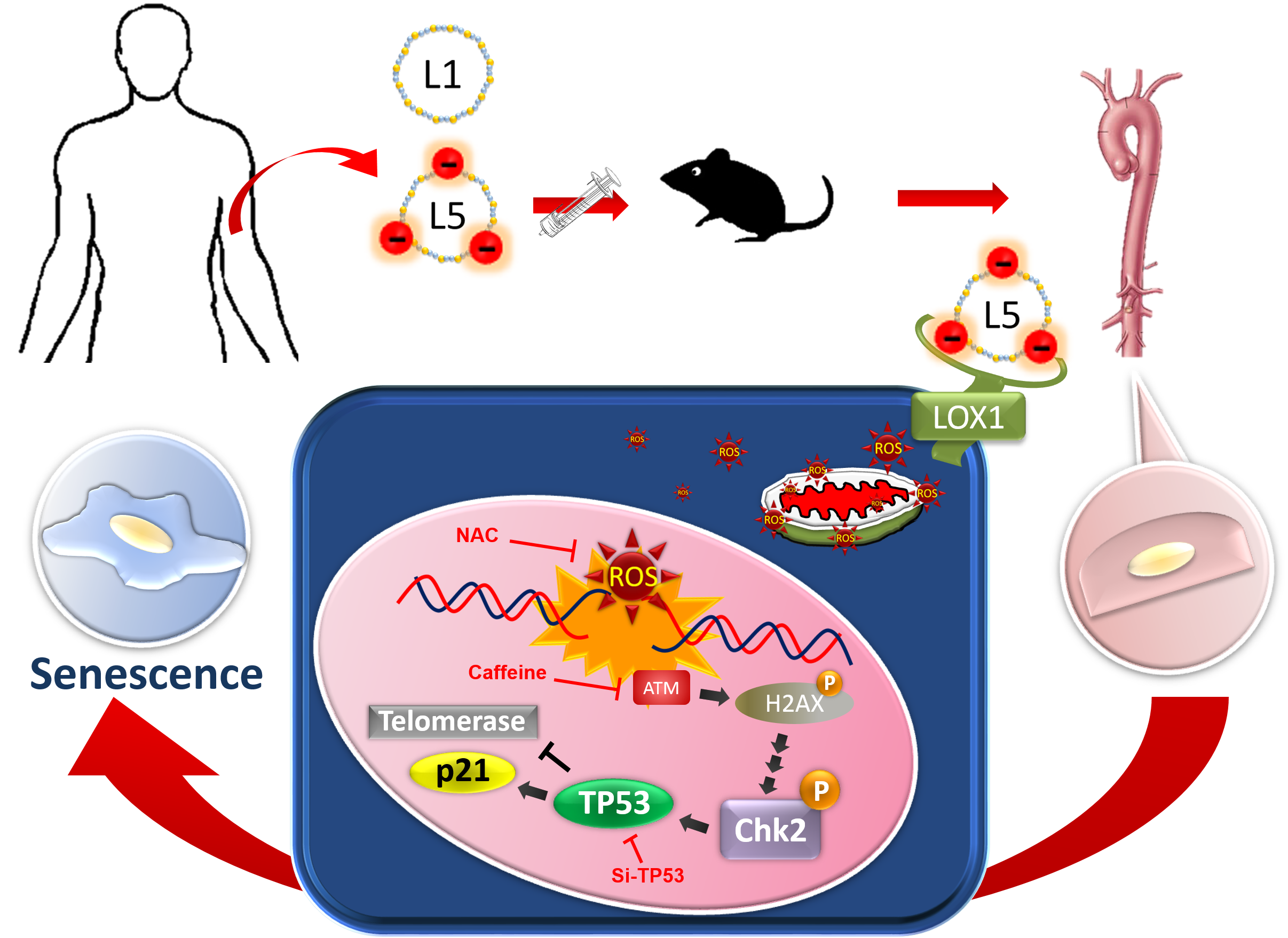

Supplement: Supplementary file 1 [file ACEL-17-na-s001.docx]
